# The Adsorption of Pb(II) from Aqueous Solution Using KOH-Modified Banana Peel Hydrothermal Carbon: Adsorption Properties and Mechanistic Studies

**DOI:** 10.3390/ma17020311

**Published:** 2024-01-08

**Authors:** Tao Bai, Jiaxin Zhao, Laixin Tian, Luming Zhang, Zhiping Jin

**Affiliations:** School of Electric Power, Civil Engineering and Architecture, Shanxi University, Taiyuan 030006, China; 202223505016@email.sxu.edu.cn (J.Z.); comtianlaixin@geg.com.cn (L.T.); 202223505014@email.sxu.edu.cn (L.Z.); jinzhipingc@126.com (Z.J.)

**Keywords:** modified hydrothermal carbon, banana peel, lead ion removal, adsorption mechanism

## Abstract

Adopting banana peel as a raw material, the adsorption properties of banana peel hydrothermal carbon modified with a KOH solution for lead ions in aqueous solution were studied. The surface structure and functional groups of the modified hydrothermal carbon were analyzed by means of X-ray diffraction (XRD), scanning electron microscopy (SEM), Fourier-transform infrared (FT-IR) spectroscopy, the Brunner–Emmet–Teller (BET) method, element analysis, and Raman spectroscopy. The results showed that an adsorption capacity of 42.92 mg/g and a removal rate of 86.84% were achieved when the banana peel hydrothermal carbon was modified with a KOH solution of 0.5 mol/L, with a pH of 6 and a solid–liquid ratio of 1 g/L. The equilibrium adsorption time for lead ions in solution being adsorbed using KOH-modified hydrothermal carbon was 240 min, the adsorption process satisfied the quasi-second-order kinetic model and the Redlich–Peterson isotherm equation, and the equilibrium removal efficiency was 88.62%. The adsorption of lead ions using KOH-modified hydrothermal carbon is mainly chemical–physical adsorption.

## 1. Introduction

Heavy metals pose great hazards when they enter water bodies, with electroplating, metallurgy, mining, the chemical industry, and processing being the main sources [1,2]. The direct discharge of industrial wastewater containing heavy metals not only causes significant harm to human health but also damages the ecological environment [3,4,5,6].

Currently, the common methods for treating heavy metals include electrocoagulation, electroflotation, chemical precipitation [7], ion exchange [8], membrane filtration, photocatalysis, the use of nanotechnology, and adsorption. Based on the comparison of technical costs and economic benefits, the ranking is as follows: adsorption > ion exchange > electroflotation > membrane filtration > electrocoagulation > chemical precipitation > photocatalysis > nanotechnology. Among them, adsorption has the advantages of wide applicability and a simple preparation method, which shows it has promising application prospects.

High-temperature pyrolysis and low-temperature hydrothermal carbonization are commonly used methods for preparing activated carbon with high adsorption performance [9]. The hydrothermal carbonization process is simple to carry out and the reaction materials do not require drying treatment. The hydrothermal carbon produced is widely used for the adsorption and removal of pollutants [10,11,12]. The hydrothermal carbonization process involves the dehydration and decarboxylation of functional groups, resulting in increased carbon content and density, as well as enhanced adsorption properties [13]. At the same time, hydrothermal carbon has been shown to display high adsorption characteristics for lead and arsenic [14].

Banana, as the fifth-largest crop and the second-largest fruit in the world, accounts for about 16% of total fruit production. With the rapid growth of China’s industrialization process, banana cultivation is becoming increasingly industrialized and its production has increased rapidly, with production exceeding 12.1 million tons in 2022. Banana peels, as by-products of banana processing in the food, beverage, and chemical industries, account for 30–40% of the total weight of bananas [15] and are typical biomass waste [16]. The main problems faced by banana peels are low recovery efficiency and long-term environmental pollution from landfilling. Studies have shown that the preparation of high-value hydrothermal carbon from banana peels using the hydrothermal method [17] exhibits good adsorption performance for lead and arsenic [16,18,19]. In a previous study, a batch experiment removed Pb(II) through a derived powered adsorbent from the banana peel.Parameters such as pH, dosage, time, and agitating speed were evaluated, and a maximum capacity of 2.18 mg/g was observed after adsorption [20]. The adsorption capacity of Pb(II) on biochar prepared from banana peel after acid treatment was increased to 20.97 mg/g [21]. Banana peel biochar was also modified and used to remove As(V), and the maximum adsorption capacity was found to be 1.04 mg/g [22]. To further improve the adsorption performance of hydrothermal carbon, modification is an effective method to change the surface activity and affinity [23]. However, there have been few studies on the surface modification of banana peel hydrothermal carbon to improve functional groups and surface affinity [24].

Based on this, the effect of different concentrations of the KOH solution, pH values, solid–liquid ratios, and adsorption times on the removal performance of lead ions in solution using hydrothermal carbon modified with KOH solution at 240 °C was studied. The surface morphology of the modified hydrothermal carbon was analyzed using XRD, SEM, FT-IR, and BET analyses and Raman spectroscopy to investigate the adsorption mechanism. This study aims to provide basic data and a theoretical basis for the industrial application of KOH-solution-modified hydrothermal carbon in removing lead ions from solution.

## 2. Materials and Methods

### 2.1. Reagents

The potassium hydroxide (KOH), xylenol orange (C_31_H_32_N_2_O_13_S), glacial acetic acid (CH_3_COOH), sodium acetate (CH_3_COONa), sulfuric acid (H_2_SO_4_), and all reagents mentioned were of analytical grade, sourced from Shanghai McClain Biochemical Technology Co., Ltd., Shanghai, China. The hydrochloric acid was of analytical grade, sourced from China Pharmaceutical Group Chemical Reagent Co., Ltd., Shanghai, China. The 1000 mg/L lead standard solution was sourced from Shanghai McClain Biochemical Technology Co., Ltd., Shanghai, China.

### 2.2. Experimental Methods

#### 2.2.1. Preparation of Banana Peel Hydrothermal Carbon

The banana peel purchased from the market was washed with tap water, dried in a 105 °C drying oven until a constant weight was achieved, and then crushed and passed through an 80-mesh sieve. It was placed in a reaction vessel, and hydrothermal carbonization was performed under N_2_. The temperature was increased from room temperature to 240 °C at a rate of 10 °C/min, and the sample was incubated for 120 min. It was then naturally cooled to room temperature, and the hydrothermal product was placed in a drying oven until a constant weight was achieved, ground again, and passed through a 70-mesh sieve to obtain banana peel hydrothermal carbon. The hydrothermal carbon prepared at 240 °C was labeled as HTC240.

#### 2.2.2. Preparation of KOH-Modified Hydrothermal Carbon

The prepared hydrothermal char (8.00 ± 0.01 g) was mixed with different concentrations of KOH solution (0.05 mol/L, 0.1 mol/L, 0.5 mol/L, and 1.0 mol/L). After being immersed in a thermostatic water bath shaker at 45 °C and 200 rpm for 6 h, the solid–liquid mixture was poured into a vacuum filtration device and washed repeatedly with anhydrous ethanol and deionized water. The solid phase was collected and dried in a constant temperature oven at 105 °C for 12 h. After that, the samples were ground and sifted through a 70-mesh sieve and placed into a sealed bag for later use. The prepared hydrothermal carbons were labeled as K-HTC-0.05, K-HTC-0.1, K-HTC-0.5, and K-HTC-1.0 according to the concentration of modified KOH solution.

#### 2.2.3. Adsorption Experiment

The stock solution of Pb(II) ions was diluted with ultrapure water to obtain the working solutions of Pb(II) ions at 50 mg/L, 100 mg/L, and 400 mg/L concentrations. The prepared 100 mg/L lead (II) stock solution was diluted to 5 mg/L, 10 mg/L, 20 mg/L, and 50 mg/L through the use of a pipette. Ultrapure water was used as a blank control, and the maximum absorption wavelength was set to 575 nm to draw the standard curve of lead ions. In this study, a UV–Vis spectrophotometer was used to measure the concentration of lead solution. The principle of the experiment is to use the different refractive indices of solutions of different concentrations to carry out quantitative measurements of lead. In the experiment, xylenol orange was used as a color reagent, and the red chelate was formed between Pb(II) and xylenol orange solution by adding the proper amount of acetic acid–sodium acetate buffer. A UV–Vis spectrophotometer was used for the quantitative determination of lead before the start of the experiment. Approximately 0.05 g (±0.001 g) of hydrothermal carbon was added to 50 mg/L lead stock solution, which was sealed and then adsorbed in a thermostatic water bath oscillator. Following the end of oscillation, it was left to stand still, and 10 mL of the upper layer of the clear liquid was placed into a centrifuge tube. After centrifugation, the absorbance of the clear liquid was measured using a spectrophotometer. The adsorption capacity and removal rate of the hydrothermal carbon were calculated from the standard curve of the lead solution according to Equations (1) and (2). All of the experiments were performed in triplicate, and results were the average of the three experiments.

(1) $$q_{e}=((C_{0}-C_{e,t})\times V)/m$$

(2) $$\eta =(C_{0}-C_{e,t})/C_{0}\times 100\%$$

where *q_e_* is the adsorption amount in mg/g; *η* is the removal rate in %; *C*_0_ and *C_e,t_* represent the initial concentration and the adsorbed concentration in mg/L, respectively; *V* is the volume of the solution in L; and *m* is the mass of the modified hydrothermal carbon in g.

The desorption experiments were designed to study the adsorption performance of the adsorbent materials for Pb(II). The modified banana peels that had reached adsorption saturation were desorbed with 0.1 mol/LH_2_SO_4_ solution and the material was placed in a constant temperature oven at 80 °C for 8 h after the experiment was repeated thrice to reach a constant weight state. It was prepared for use in the second adsorption process, and the experiment was repeated five times to illustrate the adsorption effect of recycling.

#### 2.2.4. Experimental Equipment

The pH of the solution was measured through the use of a PHSJ-3F pH meter (accuracy: 0.1, Shanghai Yidian Scientific Instrument Co., Ltd., Shanghai, China). The concentration of lead in the solution was determined through the use of a P4 UV–Vis spectrophotometer (Shanghai Meipuda Instrument Co., Ltd., Shanghai, China). The surface morphology of the hydrothermal carbon before and after modification was observed through the use of a scanning electron microscope (Teske (China) Co., Ltd., Shenzhen, China) with an acceleration voltage of 10 kV and gold spray treatment. The physical properties of the hydrothermal carbon surface before and after modification were analyzed using a Panalytical Empyrean X-ray diffractometer (China Malvern Instruments Co., Ltd., Shanghai, China). Fourier-transform infrared (FTIR) spectrometer (Somerset Fisher Technology (China) Co., Ltd., Shanghai, China) was used to analyze the types of major functional groups in the samples to further determine the structures of the substances. The pore structure of the hydrothermal carbon was measured by means of the BET method with a Micromeritics ASAP 2460 automatic specific surface and porosity analyzer, a Raman spectrometer (LabRam HR Evolution), and an element analyzer (Elementar Vario EL).

## 3. Results and Analysis

### 3.1. Surface Structure and Performance Analysis of Hydrothermal Carbon before and after Modification

#### 3.1.1. SEM Analysis

**Figure 1:**
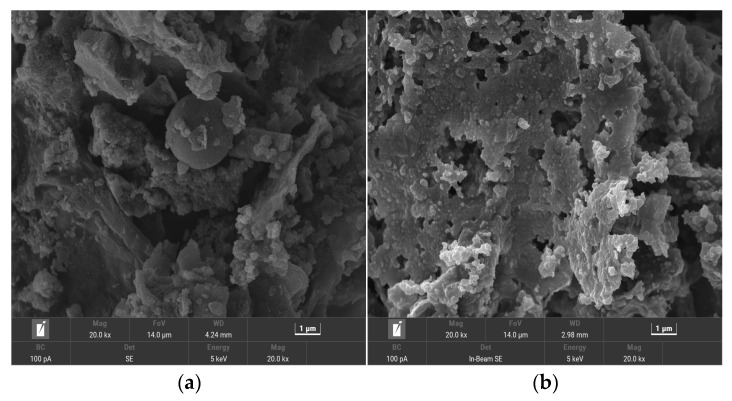

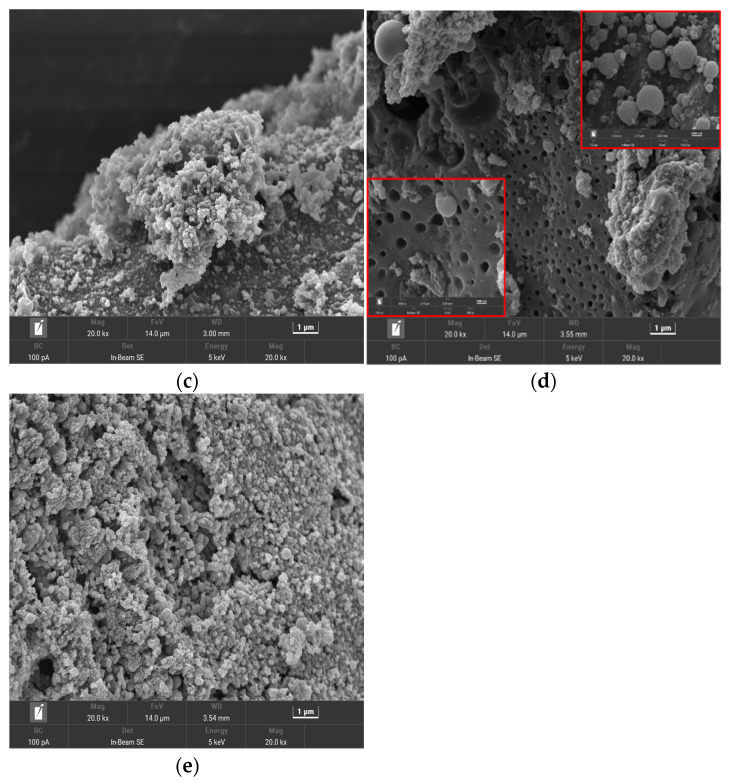
SEM images of hydrothermal carbon before and after modification: (**a**) unmodified hydrothermal carbon; (**b**) hydrothermal carbon modified with 0.05 mol/L KOH; (**c**) hydrothermal carbon modified with 0.1 mol/L KOH; (**d**) hydrothermal carbon modified with 0.5 mol/L KOH; (**e**) hydrothermal carbon modified with 1.0 mol/L KOH.

Figure 1 shows the SEM images of the hydrothermal carbon before and after modification with the KOH solution. In Figure 1a, the unmodified hydrothermal carbon has a smooth surface and fewer carbon microspheres. In Figure 1b, the surface of the hydrothermal carbon is relatively smooth with fewer and unevenly sized pores, which is due to the low concentration of KOH not causing significant changes in the surface structure. In Figure 1c of Figure 1, as the concentration of the modification solution increases, the surface of the hydrothermal carbon begins to become rough and there is a tendency for microspheres to form [25]. In Figure 1d, with increasing concentration, the surface of the hydrothermal carbon develops many microsphere carbon particles and a large number of uniformly arranged pore structures. This is because cellulose, hemicellulose, lignin, and other components in the carbon material were cracked into small carbon chain molecules under the action of KOH, and then they re-aggregated to form microspheres. The carbon pores were formed by the shown reaction in Equation (3). In Figure 1e, the surface of the hydrothermal carbon was very rough, and the pores were blocked by the generated carbon particles, which was not conducive to the smooth progress of physical adsorption.

(3) $$6\mathrm{KOH}+2C\to 2K+3H_{2}+2K_{2}CO_{3}$$

#### 3.1.2. FT-IR Analysis

**Figure 2:**
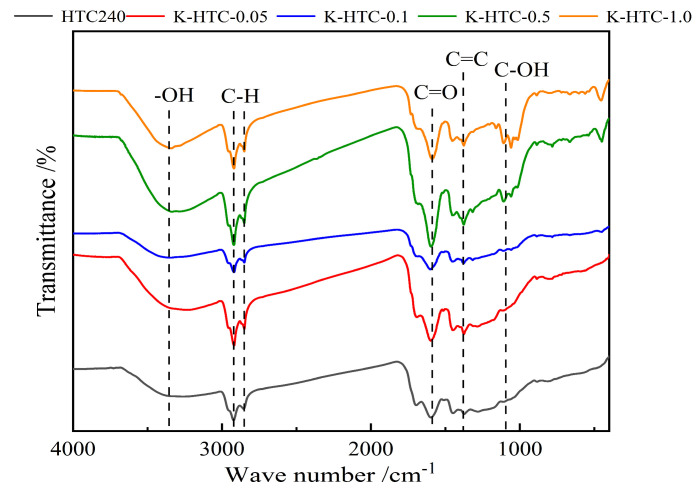
FT−IR plots of the hydrothermal carbon before and after modification.

The FT-IR spectra of the hydrothermal carbon before and after modification are shown in Figure 2. From the figure, it can be seen that the types of functional groups in hydrothermal carbon are basically the same under different conditions but there are some differences in the content. The infrared spectrum shows obvious absorption peaks at 3223 cm^−1^, 2918 cm^−1^, 2850 cm^−1^, 1600 cm^−1^, 1511 cm^−1^, 1449 cm^−1^, 1375 cm^−1^, 1328 cm^−1^, and 1059 cm^−1^. The absorption peak at 3223 cm^−1^ was attributed to the stretching vibration of -OH; the absorption peaks at 2918 cm^−1^ and 2850 cm^−1^ were attributed to the stretching vibration peaks of methyl and methylene C-H; the absorption peak at 1600 cm^−1^ was attributed to the stretching vibration of carbonyl groups (aldehydes and ketones); the absorption peaks at 1511 cm^−1^ and 1449 cm^−1^ were mainly caused by the stretching vibration of C=C, indicating the formation of hydrothermal carbon with an aromatic structure. The absorption peak at 1317 cm^−1^ was attributed to the out-of-plane bending vibration of C-H [26], and the absorption peak at 1107 cm^−1^ was attributed to the stretching vibration of C-OH.

According to Figure 2, with the increase in concentration, the peak of alcohol-OH stretching vibration after modification at 0.5 mol/L is the widest among the four conditions. This indicated that the highest hydroxyl content was produced at this concentration. The stretching vibrations of C=C, C=O, and OH first increased and then stabilized, with them reaching the maximum stretching vibration at 0.5 mol/L KOH. The stretching vibration peaks of C-O/C-O-C gradually increased, indicating that a chemical reaction occurs with potassium hydroxide, leading to a higher degree of aromatization. Aromatic carbons were more stable under both biological and non-biological oxidation conditions. Due to the aromatic structure on the surface of hydrothermal carbon, cation-π adsorption was also considered a possible mechanism for the adsorption of heavy metals using hydrothermal carbon [27].

#### 3.1.3. XRD Analysis

**Figure 3:**
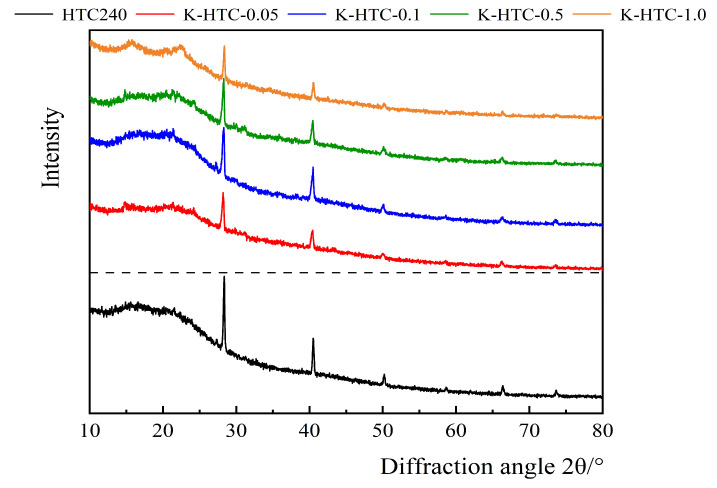
XRD patterns of the hydrothermal carbon before and after modification.

XRD characterization was performed on the hydrothermal carbons before and after modification and the results are shown in Figure 3. The characteristic diffraction peaks of potassium chloride were observed at approximately 23.1° and 42.8°. In the diffraction angle range of 10° to 30°, a broad peak was observed. With the increase in the concentration of the modifying agent, the peak area initially increased and then decreased. Carbonation was highest at 0.1 mol/L, followed by 1.0 mol/L, 0.5 mol/L, and 0.05 mol/L. An increase in the concentration of the modifying agent improved the carbonization degree of the hydrothermal carbon, suggesting that the obtained material was mainly amorphous carbon and suitable as an adsorbent material.

#### 3.1.4. BET Analysis

From the results shown in Table 1, it can be seen that the pore structure parameters of K-HTC-0.1 hydrothermal carbon are different from those of HTC240 hydrothermal carbon. K-HTC-0.1 hydrothermal carbon has a lower specific surface area and higher total pore volume and pore size than HTC240 hydrothermal carbon. According to previous studies [28], most hydrothermal carbon includes both modified and unmodified hydrothermal carbon, with specific surface areas usually between 0.6 m^2^/g and 1200 m^2^/g or even greater than 1200 m^2^/g. These values were based on the synthetic route of the adsorbent. Therefore, even some biomass adsorbents with a lower specific surface area were shown to have better adsorption potential.

K-HTC-0.1 hydrothermal carbon modified with alkaline particles on its surface can enlarge the pores of HTC240 hydrothermal carbon and increase the pore size of the hydrothermal carbon; at the same time, this will lead to a certain increase in the total pore volume. However, alkali erosivity may also cause the pore structure of HTC240 hydrothermal carbon to collapse and interconnect, resulting in a decrease in the specific surface area of the hydrothermal carbon.

#### 3.1.5. Element Analysis

The results of the element content determination of the modified and unmodified banana peels are shown in Table 2. According to the results of the element analysis, the main elements of hydrothermal carbon are C, H, O, and N, and the content of C is the highest. Comparing the contents of C, H, N, and O in the two kinds of biomass carbon, we were able to establish that the proportion of O in the KOH-modified hydrothermal carbon increased; this may be because of the increase in alcohol hydroxyl or phenol hydroxyl (-OH) groups on the carbon surface after activation.

#### 3.1.6. Raman Analysis

**Figure 4:**
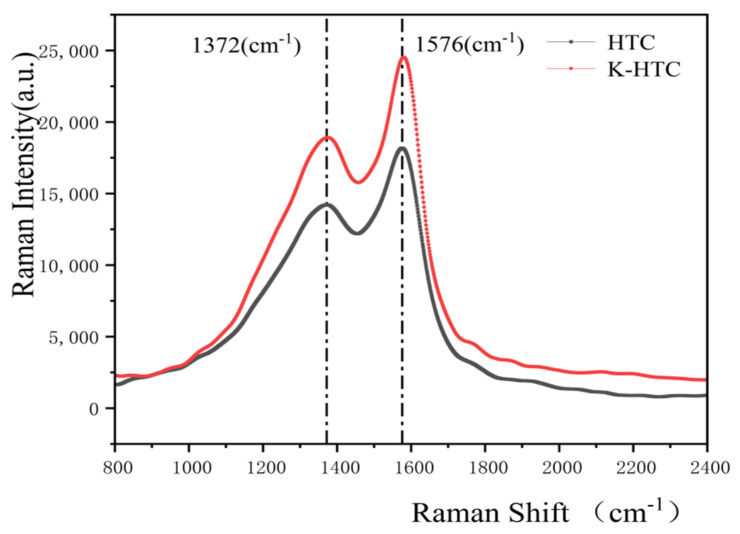
Raman spectra of the hydrothermal carbon before and after modification.

The structure defect and graphitization degree of the carbon material can be seen by the further analysis of hydrothermal carbon carried out through the use of Raman spectroscopy. Raman spectroscopy can distinguish between carbon allotropy, where the D-band may represent the sp^3^ bond (tetrahedral structure) or the orbital hybridization defect sp^2^ bond (graphene-thin edge structure) and the G-band (planar structure). The experimental results are shown in Figure 4. The prominent peak at 1576 cm^−1^ is produced by Tunku Abdul Rahman’s active vibrational mode, called the G-peak, which reflects the degree of carbon atoms hybridized by hydrothermal carbon in the sp^2^ mode, and the peak at 1372 cm^−1^ corresponds to the D-peak; in addition, the aromatization and graphitization structure of the reaction hydrothermal carbon were studied. The D-peak strength of the carbon material is lower than the G-peak, so the crystal defect degree of the carbon material is lower. In addition, the modification of the material has some influence on the disorder degree and defect degree of the carbon atomic structure, which increases the defect of the banana peel porous carbon material to a certain extent.

### 3.2. Analysis of the Lead Ion Adsorption Performance of Hydrothermal Carbon

#### 3.2.1. Influence of KOH Concentration on the Adsorption Performance of Hydrothermal Carbon

Different concentrations of KOH solution were used to modify the hydrothermal carbon derived from banana peel. The experimental conditions were a neutral solution, an adsorption time of 120 min, a temperature of 25 °C, a solid–liquid ratio of 1 g/L, and an initial concentration of 50 mg/L. The adsorption capacity and removal efficiency were calculated through the use of Formulas (1) and (2).

**Figure 5:**
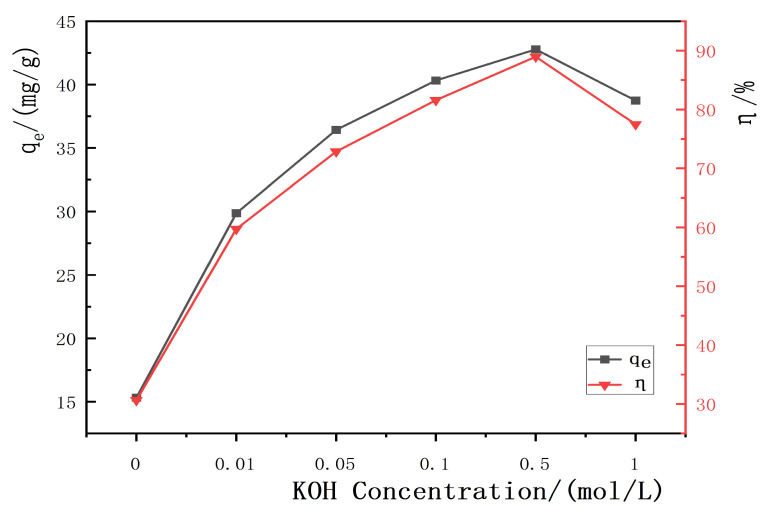
Effect of potassium hydroxide concentration on the adsorption performance and yield of the hydrothermal carbon before and after modification.

Figure 5 shows the effect of KOH concentration on the adsorption performance and yield of the hydrothermal carbon. From the graph, it can be observed that the adsorption capacity and removal rate of KOH-modified hydrothermal carbon initially increases and then decreases. The maximum adsorption capacity is achieved when the hydrothermal carbon is modified with a concentration of 0.5 mol/L KOH solution, with an adsorption capacity of 42.78 mg/g, which is 3.2 times higher than that of the unmodified hydrothermal carbon (15.32 mg/g). The yield of modified hydrothermal carbon decreased with the increase in KOH concentration. This was because the strong alkali KOH continued to hydrolyze cellulose, hemicellulose, and lignin in the banana peels as the KOH concentration increased, resulting in a rapid decrease in carbon yield. KOH could also corrode the surface of hydrothermal carbon and open up the blocked pores by means of tar deposition. The chemical reaction outlined in Equation (3) led to the destruction of the carbonaceous surface structure by KOH and the formation of new pore structures on the surface [29]. This was further confirmed by the SEM analysis, the results of which are shown in Figure 1d. Considering the higher adsorption capacity of modified hydrothermal charcoal for lead ions in 0.5 mol/L KOH solution, the subsequent adsorption experiments were conducted on the basis of this standard concentration.

#### 3.2.2. Effect of pH on Lead Ion Adsorption Performance

**Figure 6:**
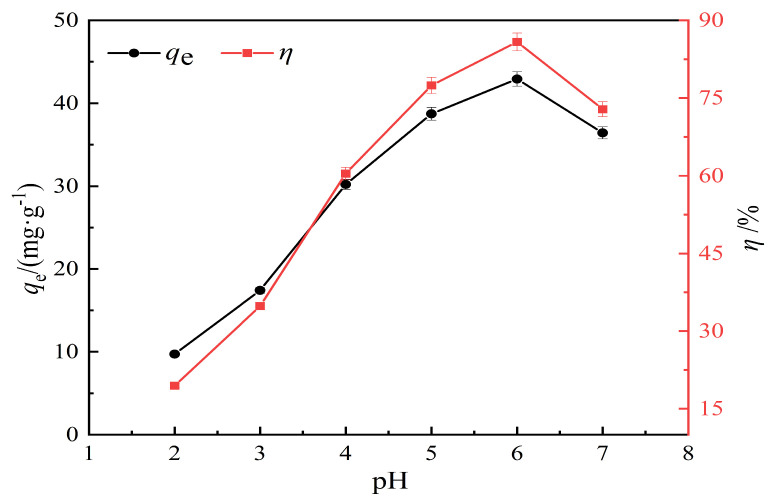
Effect of pH on Pb(II) adsorption by hydrothermal carbon.

The experimental conditions include a pH range of 2 to 7, an adsorption time of 120 min, a temperature of 25 °C, a solid–liquid ratio of 1 g/L, and an initial concentration of 50 mg/L of Pb(II) solution. Figure 6 shows the adsorption results for KOH-modified hydrothermal carbon with a concentration of 0.5 mol/L under different pH conditions.

From the results shown in Figure 6, it can be seen that the modified hydrothermal carbon exhibits an upward trend followed by a downward trend as the pH value increases. When the solution pH is 2, the adsorption capacity is 9.72 mg/g and the removal rate is 19.44%; when the pH value increases from 3 to 6, the removal rate increases significantly. At pH = 6, the adsorption capacity reaches a peak at 42.92 mg/g with a removal rate of 86.84%; when the pH is 7, the adsorption capacity of the modified hydrothermal carbon decreases. This was because when the H^+^ concentration in the solution was too high, there was a competitive relationship between [Pb(H_2_O)_6_]^2+^, Pb^2+^, and H^+^. The higher the H^+^ concentration, the more intense the competition with Pb(II). The inability of Pb(II) to bind to the active sites on the modified hydrothermal carbon prevented the adsorption from proceeding smoothly. Combined with Figure 2, it could be seen that the surface of the hydrothermal carbon contained a large number of oxygen-containing functional groups. The high H^+^ concentration caused the protonation of functional groups, which prevented the adsorption of positively charged Pb(II) cations due to electrostatic repulsion [30].

#### 3.2.3. Effect of Adsorption Time on the Adsorption Performance of Hydrothermal Carbon

**Figure 7:**
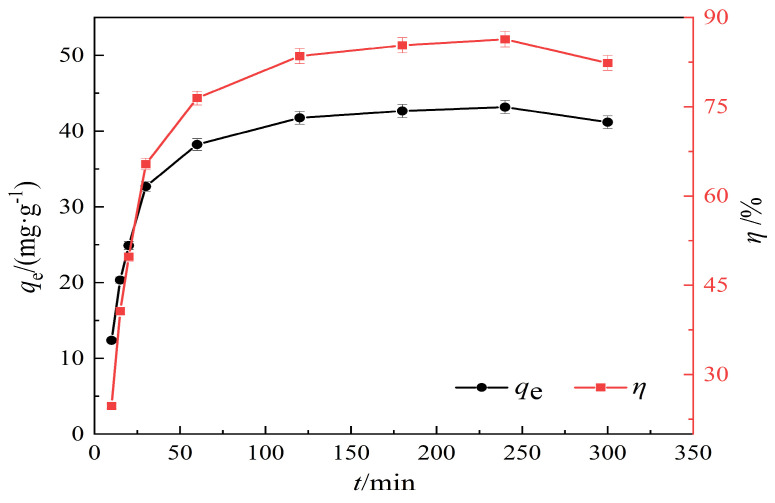
Effect of adsorption time on the adsorption of Pb(II).

The experimental conditions are pH = 7, an adsorption time ranging from 0 to 5 h, a temperature of 25 °C, a solid–liquid ratio of 1 g/L, and an initial concentration of 50 mg/L. The effect of adsorption contact time on Pb(II) adsorption is shown in Figure 7.

According to the results shown in Figure 7, it can be seen that before 120 min, the modified hydrothermal carbon exhibits a linear increase in adsorption capacity for Pb(II). With the increase in adsorption time, the adsorption rate of the modified hydrothermal carbon gradually decreases. After 240 min, the increase in adsorption rate slows down, and the removal efficiency tends to stabilize at 88.62%, with it reaching saturation. The results indicated that the adsorption process of the modified hydrothermal carbon mainly occurred within the first 120 min, with the adsorption rate being greater than the desorption rate, allowing for a rapid adsorption process. After 240 min, the decrease in Pb(II) concentration and the reduction in adsorption sites greatly reduced the probability of collision between them, which was unfavorable for adsorption [31].

#### 3.2.4. Effect of Lead Solution at Different Concentrations on the Adsorption Properties of Hydrothermal Carbon

**Figure 8:**
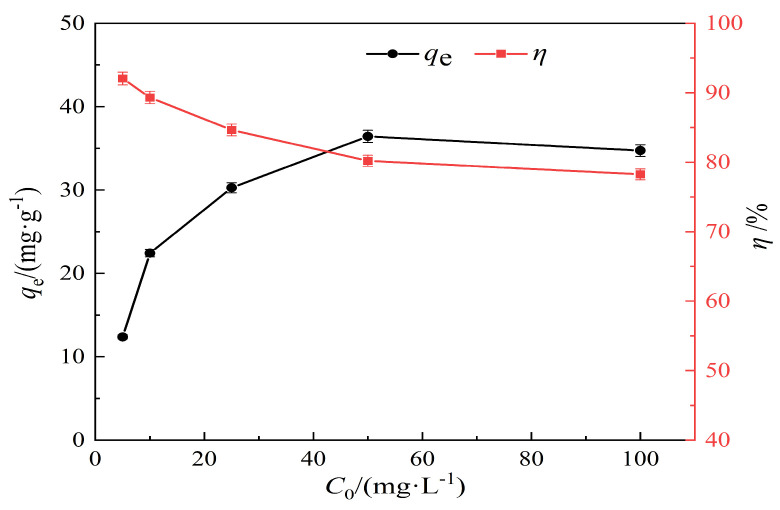
Effect of initial concentration on the Pb(II) adsorption performance of the hydrothermal carbon.

In this experiment, Pb(II) ion solutions at the concentrations of 5 mg/L, 10 mg/L, 25 mg/L, 50 mg/L, and 100 mg/L were selected. Experimental conditions: pH = 7; an adsorption time of 120 min; a temperature of 25 °C; a solid–liquid ratio of 1 g/L; and hydrothermal carbon modified by KOH of 0.5 mol/L concentration. The effect of the initial concentration on Pb(II) adsorption using modified hydrothermal carbon is shown in Figure 8.

As shown by the results displayed in Figure 8, with the increase in initial Pb(II) concentration, the adsorption capacity of modified hydrothermal carbon for Pb(II) shows a trend of linear increase. When the initial concentration of lead solution is 5 mg/L, the removal rate can reach 92.05%. With the increase in initial concentration, the removal rate decreases significantly to 78.26% at 100 mg/L. This was because at lower initial concentrations, the affinity of the adsorbent for the adsorbate was high and the content of Pb(II) was low, which was conducive to the adsorption of Pb(II) [32]. With the increase in initial concentration, the concentration of Pb(II) increased, and the driving force of concentration made the adsorption sites move towards the interior of the pores, resulting in an increase in the adsorption capacity [33].

#### 3.2.5. Adsorption Kinetics of Modified Hydrothermal Carbon

To analyze the variation in the hydrothermal carbon adsorption process over time and provide evidence of the adsorption mechanism, the first-order kinetics, the second-order kinetics, and the three Elovich models were fitted. The model formulas are shown in Equations (4)–(6). The experimental conditions were a neutral solution, an adsorption time of 0–6 h, a temperature of 25 °C, a solid–liquid ratio of 1 g/L, and an initial concentration of 50 mg/L.

(4) $$q_{t}=q_{e}\times [1-\exp(-k_{1}\times t)]$$

(5) $$q_{t}=(k_{2}\times {q_{e}}^{2}\times t)/(1+k_{2}\times q_{e}\times t)$$

(6) $$q_{t}=(1/\beta )\times \ln(\alpha \times \beta )+(1/\beta )\times \ln(t)$$

According to Table 3, it can be seen that the correlation coefficient R^2^ after fitting the second-order kinetics is 0.98, which is significantly higher than the fitting effect of the other two models. The calculated adsorption capacity (45.48 mg/g) of the second-order kinetics model for modified hydrothermal carbon is close to the actual adsorption capacity (44.61 mg/g), with a relative error of 1.9%. According to the modeling principles of the second-order kinetics model, this indicates that the adsorption process is complex, with different situations such as external liquid film diffusion, surface adsorption, and intra-particle diffusion [34]. This is because the second-order kinetics equation can well describe the adsorption process of modified hydrothermal carbon and produce similar results to isotherm simulation, indicating a chemical–physical adsorption process.

#### 3.2.6. Modified Hydrothermal Carbon Isothermal Adsorption Analysis

Under parallel conditions of 293 K, 303 K, and 313 K, the initial solution concentration was 5–500 mg/L, pH = 7, the adsorption time was 120 min, the solid–liquid ratio was 1 g/L, and the concentration of hydrothermal carbon modified by KOH was 0.5 mol/L. The isotherm model was fitted using the measured adsorption capacity.

The Langmuir isotherm model and the Freundlich isotherm model are the two dynamic models used in this study. The Langmuir isotherm model simulates that the adsorbent is a monolayer adsorbent on the surface of a homogeneous adsorbent, and there is no interaction force between the adsorbent and the molecule; it is a nonlinear formal equation. The specific relationship is shown by Equations (7) and (8):

(7) $$q_{e}=(q_{m}\times K_{L}\times C_{e})/(1+K_{L}\times C_{e})$$

(8) $$R_{L}=1/(1+K_{L}\times C_{e})$$

where *q_e_* denotes the adsorbent concentration in the solid phase at equilibrium, mg/g; *q_m_* denotes the maximum adsorption capacity, mg/g; *K_L_* denotes the adsorption constant, L/mg; *C_e_* denotes the adsorbent concentration in the liquid phase at equilibrium, mg/L; *C*_0_ denotes the initial concentration of the adsorbent, mg/L; and R_L_ denotes the separation factor.

The Freundlich isotherm model can be applied to multilayer adsorption. The affinity of the adsorbent is not uniformly distributed on the heterogeneous surface, and the adsorption capacity is the sum of the capacities of all adsorption sites. The specific relationship is shown by Equation (9):

(9) $$q_{e}=K_{F}\times {C_{e}}^{1/n}$$

where *C_e_* represents the adsorbent concentration in the liquid phase at equilibrium, mg/L; *K_F_* represents the adsorption constant; *q_e_* represents the adsorbent concentration in the solid phase at equilibrium, mg/g; and *n* represents a dimensionless parameter.

The Redlich–Peterson model is a hybrid isotherm model that features the Freundlich and Langmuir isotherm models with three parameters [35]. The specific relationship is shown by Equation (10):

(10) $$q_{e}=\frac{K_{R}C_{e}}{1+a_{R}{C_{e}}^{g}}$$

where *C_e_* represents the adsorbent concentration in the liquid phase at equilibrium, mg/L; *q_e_* represents the adsorbent concentration in the solid phase at equilibrium, mg/g; *K_R_* denotes the adsorption constant; and a_R_ represents a dimensionless parameter.

**Figure 9:**
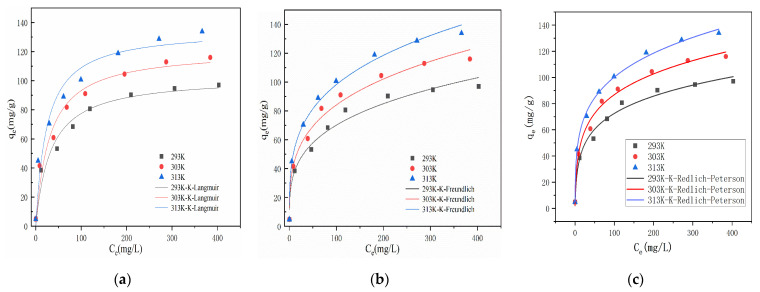
Adsorption isotherm of Pb(II) on hydrothermal carbon: (**a**) the Langmuir isotherm model; (**b**) the Freundlich isotherm model; (**c**) the Redlich–Peterson model.

From the results shown in Figure 9, it can be seen that the adsorption capacity of hydrothermal carbon for Pb(II) increases from 4.85 mg/g to 119.31 mg/g at 293 K when the adsorption equilibrium is in the range of 5–200 mg/L. The adsorption capacity increases rapidly, exhibiting the typical characteristics of chemical adsorption [36]. With the increase in initial concentration, the adsorption capacity increased slowly, and the active sites on the surface of the hydrothermal carbon transited from an unsaturated state to a saturated state, eventually reaching adsorption equilibrium. The adsorption capacity also varied at different temperatures, with the adsorption capacity of the hydrothermal carbon gradually increasing as the temperature rose. The analysis results suggest that the increase in temperature facilitates the Brownian motion of molecules, increases the effective collision between Pb(II) and surface active functional groups, and promotes molecules to enter deeper pore structures, thereby enhancing the adsorption capacity. This indicates that higher temperatures were beneficial for smooth adsorption [37].

Table 4 shows the isotherm constants for adsorption. The adsorption of Pb(II) on modified hydrothermal carbon was fitted using Langmuir, Redlich–Peterson, and Freundlich models at temperatures of 293 K, 303 K, and 313 K. The correlation coefficients (R^2^) for the Langmuir model were 0.96, 0.96, and 0.95, respectively. The correlation coefficients were greater than or equal to 0.95, and the KL values were less than 1, indicating that adsorption was carried out with ease and the adsorption process was irreversible chemisorption [38]. For the Freundlich model, the corresponding R^2^ values were 0.97, 0.98, and 0.98 at 293 K, 303 K, and 313 K. The correlation coefficients were greater than 0.95, which was higher than that of the Langmuir model. This indicated that the “active sites” on the surface of the modified hydrothermal carbon were randomly distributed, suggesting differences in adsorption capacity and multilayer physical adsorption. When the value of 1/n in the Freundlich model was less than 1, it could be considered that the adsorption process was favorable under the studied experimental conditions. The analysis showed that during the adsorption process of the modified hydrothermal carbon, Pb(II) first bound to the active sites on the surface and chemisorption occurred. As the reaction proceeded, the number of active sites decreased and the driving force for chemisorption was weakened. The scanning electron microscopy analysis results displayed in Figure 1 show that the modified hydrothermal carbon has a strong porous structure, and under the driving force of physical adsorption, Pb(II) continuously filled the pores and underwent internal diffusion to adsorb onto the new active sites on the modified hydrothermal carbon, resulting in multilayer physical adsorption. For the Redlich–Peterson model, the corresponding R^2^ values at 293 K, 303 K, and 313 K were 0.98, 0.99, and 0.99, respectively, with higher correlation coefficients than those of the other two models, so that the adsorption process could be better fitted. Since this model was a combination of the Freundlich and Langmuir models, the adsorption mechanism did not follow the ideal monolayer adsorption. It was linear with concentration and represented adsorption equilibrium over a wide range of concentrations [39].

### 3.3. The Reusability of Hydrothermal Carbon

**Figure 10:**
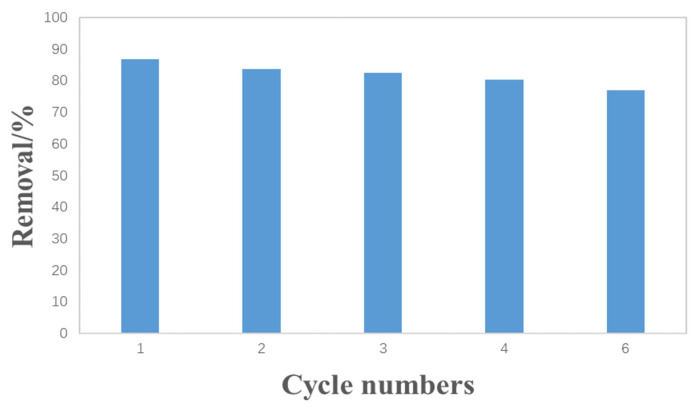
Removal efficiency of Pb(II) over five cycles of hydrothermal carbon.

Considering its practical application, if the adsorption material can be used repeatedly, the cost of sewage treatment can be effectively reduced. In order to study the cyclic adsorption of Pb(II) on hydrothermal carbon, the modified banana peel hydrothermal carbon was dried and desorbed after reaching saturation adsorption under the optimum adsorption conditions. Figure 10 shows the removal rate of Pb(II) during each cycle after the desorption of H_2_SO_4_ solution at a concentration of 0.1 mol/L. According to the graph, after five cycles, the removal rate of Pb(II) decreased slightly from 86.84% to 76.88%.

## 4. Conclusions

In this work, the physicochemical properties of potassium hydroxide-modified banana peel were studied by means of XRD, SEM, FT-IR spectroscopy, the BET method, element analysis, and Raman spectroscopy. The effects of the pH value, adsorption time, solid–liquid ratio, and initial concentration of potassium hydroxide on the adsorption properties of modified hydrothermal carbon were studied. The adsorption mechanism of modified hydrothermal carbon on Pb(II) was studied by means of adsorption thermodynamics and kinetic simulation. The following conclusions were reached:

(1) K-HTC is a carbon material obtained by KOH modification of banana peel hydrothermal carbon. This mainly included amorphous carbon with a large number of oxygen-containing functional groups on its surface, which could provide active sites for adsorption;
(2) An adsorption capacity of 42.92 mg/g and a removal rate of 86.84% were achieved when the banana peel hydrothermal carbon was modified with a KOH solution with a pH of 6 and a solid–liquid ratio of 1 g/L;
(3) The Redlich–Peterson model in the adsorption thermodynamics model was more suitable for the adsorption process of modified hydrothermal carbon on Pb(II), with correlation coefficients (R^2^) of 0.98, 0.99, and 0.99 at 293 K, 303 K, and 313 K, respectively, indicating a multilayer adsorption process. The second-order kinetic model in the adsorption kinetics model better represented the adsorption process of modified hydrothermal carbon on Pb(II), indicating chemical–physical adsorption;
(4) Hydrothermal carbon is a new type of adsorbent with the advantages of high adsorption capacity, good adsorption effects, and simple regeneration. The hydrothermal carbon method could effectively aid in removing heavy metal pollutants in wastewater and has a wide range of application prospects.

## Figures and Tables

**Table 1:** Pore structure parameters of the modified hydrothermal carbon.

	BET Surface Area /(m^2^∙g^−1^)	Total Pore Volume /(cm^3^∙g^−1^)	Average Pore Size /nm
HTC240	7.3709	0.026279	14.2607
K-HTC-0.1	5.6700	0.034832	24.5729

**Table 2:** Content of C, H, O, and N in biomass carbon.

	C/%	H/%	N/%	O/%
HTC240	69.688	6.229	3.107	15.730
K-HTC-0.1	66.832	5.928	2.843	18.029

**Table 3:** Dynamic equation parameters and correlation coefficient table.

Type of Dynamic Model	Parameters and Correlation Coefficients			
First-order kinetics	*k*_1_	*q_e_*	h_0_	R^2^
	1/min	mg/g	mg/(min∙g)	
	4.45	42.03	187.03	0.91
Second-order kinetics	*k*_2_	*q_e_*	h_0_	R^2^
	g/(mg∙min)	mg/g	mg/(min∙g)	
	0.14	45.48	289.58	0.98
Elovich	*α*	*β*	-	R^2^
	1802.09	0.15	-	0.89

**Table 4:** Table of the correlation coefficients of adsorption isotherm parameters.

Isotherm Model	Experimental Conditions	Parameters and Related Parameters			
Langmuir	T/K	K_L_/L∙mg^−1^	q_m_/mg∙g^−1^	-	R^2^
	293	0.036	101.27	-	0.96
	303	0.044	120.19	-	0.96
	313	0.055	131.53	-	0.95
Freundlich	T/K	K_F_ (mg/g(1/mg)1/*n*)	1/*n*	-	R^2^
	293	19.61	0.28	-	0.97
	303	23.33	0.28	-	0.98
	313	28.27	0.27	-	0.98
Redlich–Peterson	T/K	K_R_ (mg/g(1/mg)1/*n*)	g	a_R_	R^2^
	293	14.64	0.79	0.51	0.98
	303	20.23	0.78	0.60	0.99
	313	65.57	0.77	1.88	0.99

## Data Availability

Data are contained within the article.
